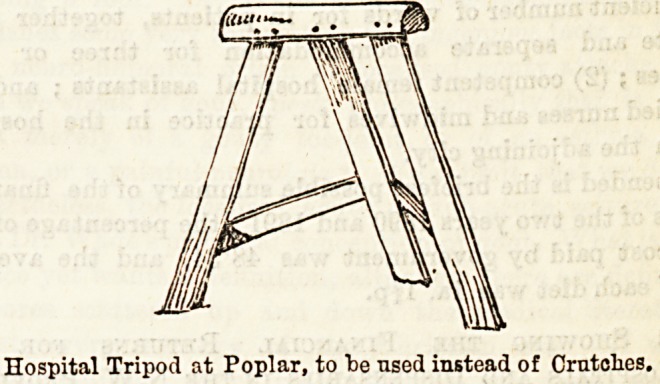# Practical Departments

**Published:** 1894-01-06

**Authors:** 


### PRACTICAL DEPARTMENTS.

### APPLIANCES AT POPLAR HOSPITAL.

BED-REST.

During a recent inspection of the Poplar Hospital for Accidents our admiration was especially excited by certain of the ward fittings and appliances, many of which were novel and much to be commended from every point of view. In a subsequent comment upon the hospital and its work (see THE HOSPITAL for October 28th, page 61) special mention was made of these, and by the kind permission of the Matron, Miss Vacher, we are now able to give a more detailed description, illustrated with sketches.

The bed-rest here pictured may claim, we believe, to be a "Poplar invention," and those supplied to the wards of the Accident Hospital are made by Messrs. McWhirter, Roberts, and Co., 249, East India Dock-road. The painted iron frame

Bed Rest at Poplar Hospital.

is made just to fit the bedstead in width, being thirty inches wide by twenty-four inches in height. As is clearly shown in the drawing, it may be raised in accordance with the patient's comfort to any height, or correspondingly lowered, by means of the hinged back, whilst the pillows are kept well in place by means of the projecting arms, which can also be folded away if preferred.

The canvas back is firm, without being hard, and is altogether admirable for purposes of cleanliness, being easily removed for washing and disinfecting. A special advantage is the exceptionally firm support it gives—no small recommendation, as will be testified by the patients in the bright and cheery wards of the Poplar Hospital. The bed-rest is a really excellent contrivance, and cannot fail to prove a comfort and convenience wherever adopted.

TRIPOD.

This, as well as the bed-rest already described, is a Poplar speciality, and fully as much to be commended. It is really a most ingenious and helpful form of crutch, and is much appreciated by the patients, who find themselves far more able to move about with ease and confidence by its aid, than with the ordinary crutches, which on polished ward floors are more of the nature of accident traps than a safe method of locomotion.

Hospital Tripod at Poplar, to be used instead of Crutches.

With the two hands firmly grasping the top bar it is easy even for the owner of an injured limb to propel himself along, with no possible fear of a slip, and the sense of security thus given is an immense comfort, as may well be fancied. The "Tripod" is a neat-looking little affair, made in plain or polished wood, and is the work of a local carpenter. The height is 31 in., by 16½ in width. As an adjunct to an accident ward it will only need to be known for its advantages to be as fully appreciated as they deserve.

## Figures and Tables

**Figure f1:**
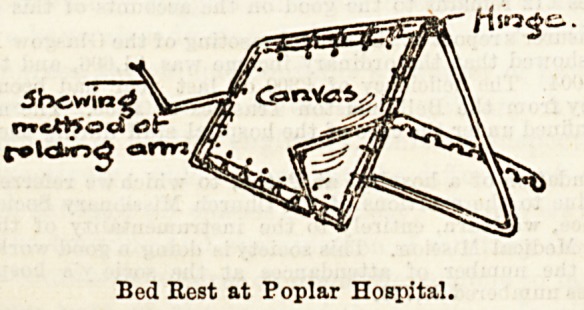


**Figure f2:**